# Bilateral and unilateral load-velocity profiling in a machine-based, single-joint, lower body exercise

**DOI:** 10.1371/journal.pone.0222632

**Published:** 2019-09-16

**Authors:** Carlos Balsalobre-Fernández, Mario Cardiel-García, Sergio L. Jiménez

**Affiliations:** 1 Department of Physical Education, Sport and Human Movement, Autonomous Univerisity of Madrid, Madrid, Spain; 2 School of Sports Science, European University of Madrid, Madrid, Spain; University of Brasilia, BRAZIL

## Abstract

**Background:**

To analyze the goodness of fit of the load-velocity relationship in a machine-based, single-joint exercise performed both in a bilateral and unilateral manner, as well as to study its accuracy to estimate one repetition maximum (1-RM).

**Methods:**

Fifteen resistance trained males performed an incremental test in the bilateral and unilateral leg extension exercise up to the 1-RM in two separate occasions. Mean vertical velocity of the weight plates in the leg extension machine was measured for every repetition using a smartphone application (My Lift).

**Results:**

Linear regression analyses showed a high goodness of fit (R^2^ > 0.93) and small standard errors of estimate (SEE < 5%1-RM) both in the bilateral and unilateral leg extension when individual load-velocity regressions for each participant were computed. Unilateral load-velocity relationships showed significant differences in the intercept of the regression line with the Y-axis and the velocity at each percentage of the 1-RM (Cohen’s *d* > 1.0, p< 0.05). Finally, non-significant differences were observed between actual and estimated 1-RM from the load-velocity relationships (*r =* 0.88.0–96, Cohen’s *d* < 0.2, p> 0.05).

**Conclusions:**

This proof of concept highlights that computing load-velocity relationships in a machine-based, single-joint, angular exercise can be appropriately performed by measuring the mean vertical velocity of the weight plates. These results could help strength and conditioning researchers and coaches who wish to analyze load-velocity relationship in other common machine-based exercises.

## Introduction

Measuring movement velocity during resistance training is known to be a non-invasive and accurate way to prescribe intensity and manage fatigue [1–4]. It has been extensively demonstrated that there is a nearly perfect association between movement velocity and the percentage of the 1-repetition maximum in different exercises, especially when individual load-velocity relationships are computed [1,3,5–7]. Therefore, movement velocity has been proposed as an alternative to traditional 1-repetition maximum (1-RM) testing, because it allows to estimate training intensity without conducting a maximal effort. Also, it has been observed that prescribing training loads based on velocity metrics, rather than using percentages of the 1-RM is more efficient to improve maximal strength and barbell kinematics [8,9]. The main problem of the so-called *velocity-based training* paradigm is that the load-velocity relationships are exercise-dependent [3,5], meaning that if movement velocity is to be used to prescribe training intensity in a certain exercise, its load-velocity relationship needs to be previously investigated. For example, it is known that lower-body exercises like the back-squat allow higher velocities at each percentage of the 1-RM in comparison with upper-body movements like the bench-press [5,6]. To date, the load-velocity relationships of several exercises like the bench-press, back squat, deadlift, hip-thrust or pull-up have been measured [1,5,10,11]. Despite most studies in the literature use barbell velocity to study the load-velocity relationships in different exercises, there is a number of investigations that analyzed the linear motion of the weight plates in machine-based exercises like the leg-press to calculate movement velocity [3,5]. For example, the load-velocity relationship in the lat-pull down and the seated cable row have been recently analyzed by recording the vertical ascent of the weight plates with a smartphone application [12], showing high levels of validity for the estimation of 1-RM [3]. Moreover, bilateral and unilateral force-velocity relationships of the leg extension exercise and its association with muscular performance have been recently analyzed by measuring the vertical ascent of the weight plates with a linear transducer [13]. However, the analysis of the load-velocity relationship in the leg extension exercise and its suitability to estimate 1-RM, both in a bilateral and unilateral manner, has not been previously investigated. This investigation aims to analyze the relationship between movement velocity and load (in terms of %1-RM) in the leg extension exercise, and to study its capacity to estimate the 1-RM. We hypothesize that there will be a very high association between velocity and load in the leg extension exercise performed in a bilateral and unilateral manner, and that there will be no statistically significant difference between actual and estimated 1-RM.

## Materials & methods

### Participants

**Fig 1:**
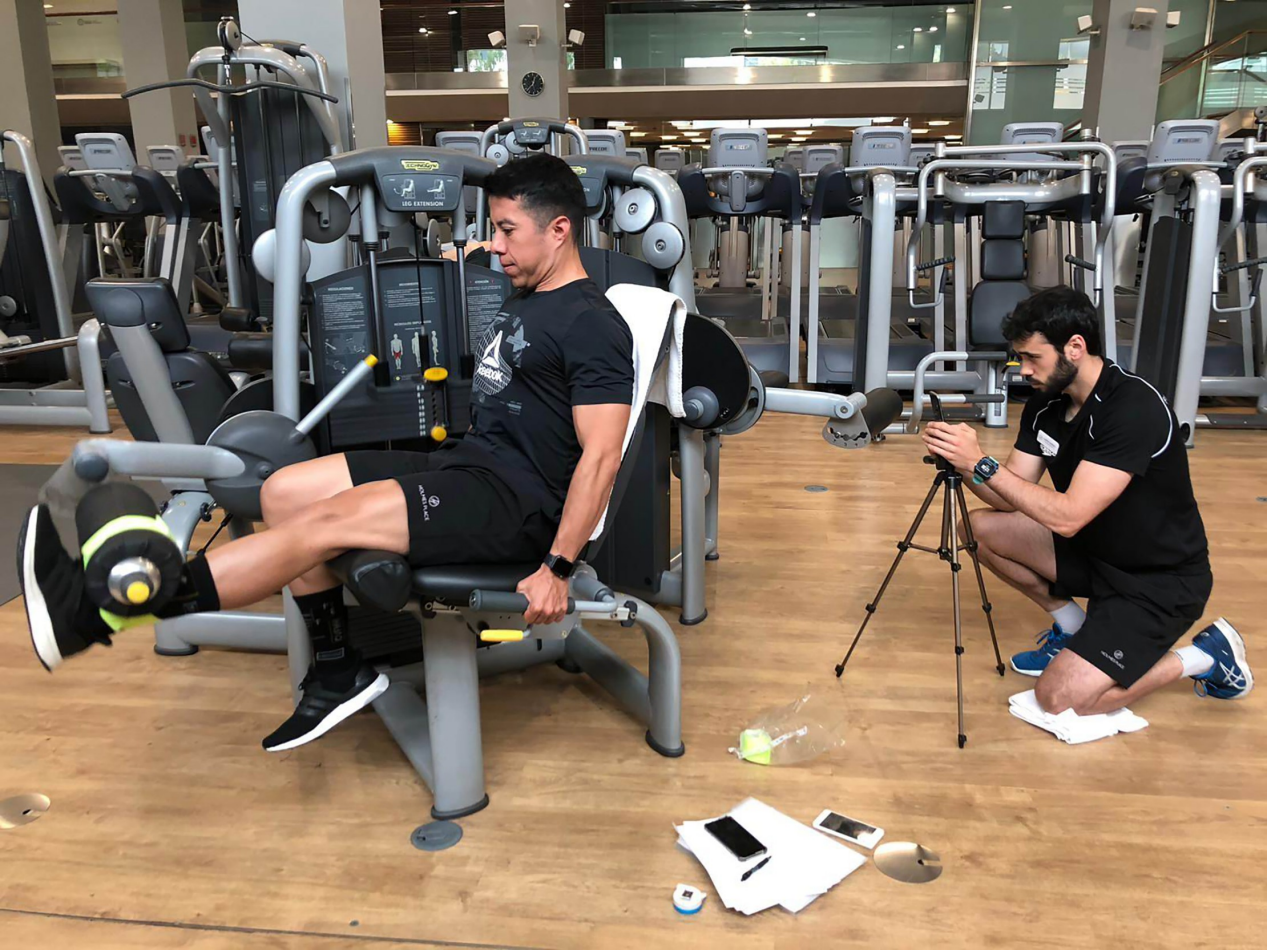
Position of the smartphone to record mean velocity of the weight plates in the leg extension machine.

Fifteen males with at least 2 years of experience in the bilateral and unilateral leg extension exercises took part in this study (N = 15; age = 33.6±9.3 years). All subjects were instructed to avoid any strenuous exercise two days before each testing session. They were informed of the study procedures and signed a written informed consent form prior to initiating the study. The study protocol adhered to the tenets of the Declaration of Helsinki and was approved by the Institutional Review Board at the European University of Madrid. The individuals in Fig 1 have given written informed consent (as outlined in PLOS consent form) to publish it in this study.

### Experimental design

A correlational design was carried out to analyze the load-velocity relationship during the bilateral and unilateral leg extension exercise in fifteen resistance trained males by recording the weight plates of the machine with a slow-motion smartphone application. Load-velocity relationships were computed, and the goodness of fit of the load-velocity relationship was analyzed by means of linear regression models as extensively reported [6,14]. Finally, individual and general load-velocity relationships (i.e., the scores obtained when computing the load-velocity relationship of each individual *vs*. when using the whole dataset combined) obtained during the bilateral and unilateral leg extension exercise were compared.

### Testing procedures

After conducting a 10-min warm-up consisting on 5 min of jogging and dynamic stretches, subjects’ one repetition maximum (1-RM) in the bilateral leg extension exercise was measured using an incremental protocol as described elsewhere [6]. One repetition per set was performed, and 3-min of passive rest were allowed between each set. The leg extension machine was adjusted so each individual could start the movement at a knee flexion of 90º. Subjects were instructed to perform each repetition at maximum intended velocity and to complete the full extension of the knee. A certified strength and conditioning coach supervised each 1-RM incremental test to ensure that subjects performed the bilateral leg extension exercise with proper technique (i.e., without any hip extension and achieving full leg extension). If a repetition did not meet the aforementioned criteria, the coach asked the participant to perform the repetition again after 1-min. of passive rest. The initial external load was set at 40 kg and 25 kg for the incremental bilateral and unilateral test (respectively) for all participants and was progressively incremented at a rate of 5-10kg until they were unable to complete another lifting. When participants failed one lift, the load was reduced in a range of 0.5 to 2.5 kg to determine their 1-RM with a high degree of precision. The number of total sets in the incremental tests ranged 4–7 sets, with an average of 5.0 ± 1.2 sets. Mean velocity during the bilateral leg extension exercise was measured for each repetition by using the validated *My Lift* v.8.1 iOS app [12], which was installed on an iPhone 8 with the iOS 12.2 operative system (Apple Inc., USA). The app was used to record the ascent of the weight plates of the leg extension machine at 240 frames per second at a quality of FullHD (1080p). The iPhone was mounted on a tripod and positioned at 1.5m from the weight plates of the leg extension machine (see Fig 1). Then, mean velocity was calculated using Eq 1 from the fundamental laws of motion:

$$v=\frac{d}{t},$$ (1)

where *v* is the mean velocity of the concentric phase of the movement, *t* is its duration and *d* is the vertical displacement of the weight plates (ROM). The app measured the duration of the concentric phase of the movement by manually selecting the frame in which the weight plates started their vertical ascent (i.e., beginning frame) and the frame in which the weight plates stopped their vertical ascent (i.e., end frame). ROM was measured with a metric tape as the distance from the rest position of the weight plates to their maximum vertical position at each subject’s full knee extension. In that final position, a mark was made in the machine using a tape. A certified strength and conditioning coach carefully supervised each repetition to guarantee that participants moved the weight plates until their individual mark (meaning that ROM was the same at each load), and if there was any doubt, the movement of the weight plates was further analyzed by visually inspecting the slow-motion video recorded with the smartphone. The use of a metric tape to measure ROM was validated and successfully implemented in different studies, both with barbell and machine-based exercises [3,12,15].

Then, the following parameters from the load-velocity relationships were computed in order to compare bilateral and unilateral tests: R^2^, the standard error of the estimate (SEE), the slope and the intercept with the Y-axis of the regression line, and the theoretical velocity at 40, 70 and 100% of the 1-RM.

After 48-h from the bilateral test, subjects followed the same procedure to measure the 1-RM of each leg in the unilateral leg extension exercise. Participants reported which leg did they felt more comfortable with when kicking a ball to register leg dominance. Then, the test was started with the dominant or non-dominant leg randomly. Each load was performed with each leg before moving to the next incremental set. Thirty seconds of passive rest were allowed between sets with each leg, and 3 minutes of passive rest were allowed until performing the next incremental load. Finally, sets below 90%1-RM were computed to create individual load-velocity relationships in order to estimate the 1-RM. Maximal loads were not included in the computation of the load-velocity relationships in order to test its estimation of the 1-RM with submaximal loads, as has been previously studied [3,16].

### Statistical analyses

Data are presented as means and standard deviations (SD), and normal distribution for all variables (Shapiro–Wilk test) and the homogeneity of variances (Levene's test) were confirmed (*p* > 0.05). The coefficient of determination (R^2^) and the standard error of the estimate (SEE) were used to assess the goodness of fit of the generalized and individualized load-velocity relationships for the bilateral and unilateral tests using a linear regression model. Linear rather than polynomic regressions have been proposed as a simpler and more reliable method to analyze the load-velocity relationship [7]. Cohen’s *d* effect size (ES) with 95% confidence intervals (CI) were used to assess the magnitude of the differences between bilateral and unilateral load-velocity relationships. The criteria for interpreting the magnitude of the ES were: trivial (<0.2), small (0.2–0.6), moderate (0.6–1.0) and large (>1.0) [17]. One-way ANOVA was used to analyze the differences between bilateral and unilateral load-velocity relationships. Finally, Pearson’s product-moment correlation coefficient, SEE, Cohen’s *d* with 95%CI and paired T-Test were computed to study the associations between actual and estimated 1-repetition maximum. The level of significance was set at 0.05. All statistical analyses were performed using JASP 0.9.2 for macOS (University of Amsterdam, Netherlands).

## Results

### Bilateral and unilateral load-velocity relationships

**Fig 2:**
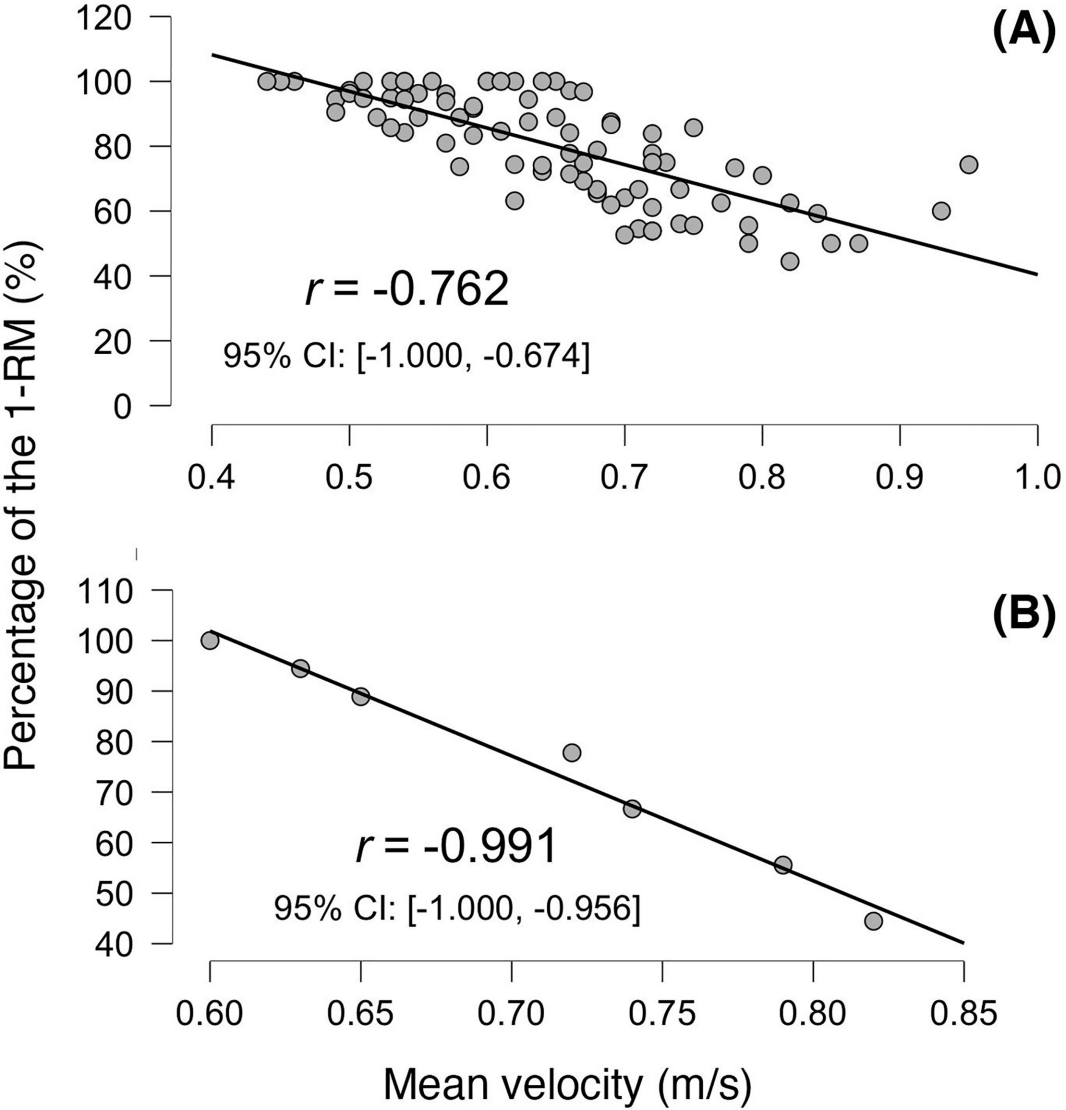
Generalized (A) and a typical load-velocity relationship in the bilateral leg extension exercise (B).

When analyzing each individual load-velocity relationship, high associations between mean velocity and load (in terms of %1-RM) were observed both for the bilateral (R^2^ = 0.96 ± 0.02; SEE = 3.60 ± 1.19%1-RM, p< 0.001) and unilateral test (R^2^ = 0.93 ± 0.07; SEE = 4.27 ± 2.44%1-RM, p< 0.001). Finally, when comparing bilateral and unilateral relationships via a one-way ANOVA, significant differences were found in the intercept of the regression line with the Y-axis (p<0.05) and the velocity associated to 40%, 70% and 100% 1-RM (p<0.05) between the bilateral test and the dominant and non-dominant legs’ tests. No significant differences were observed between dominant and non-dominant legs. See Fig 2 and Table 1 for more details.

**Table 1:** Parameters of the load-velocity relationships in the bilateral and unilateral tests.

	*Bilateral*	*Dominant leg*	*ES (95% CI)*	*Non-dominant leg*	*ES (95% CI)*
R^2^	0.96 ± 0.02	0.94 ± 0.06	-0.49 (-1.23, 0.25)	0.84 ± 0.28	0.68 (-0.12, 1.48)
SEE (%)	3.6 ± 1.19	4.3 ± 2.28	0.43 (-0.34, 1.2)	3.79 ± 2.89	-0.09 (-0.87, 0.68)
Intercept	1.13 ± 0.27	0.91 ± 0.10*	-1.02 (-1.79, -0.23)	0.82 ± 0.28*	1.11, (0.26, 1.94)
Slope	-0.005 ± 0.002	-0.004 ± 0.001	0.52 (-0.22, 1.26)	-0.004 ± 0.002	-0.67 (-1.47, 0.12)
V40 (m/s)	0.90 ± 0.16	0.73 ± 0.06*	-1.31 (-2.10, -0.49)	0.72 ± 0.05*	1.32 (0.43, 2.20)
V70 (m/s)	0.72 ± 0.09	0.59 ± 0.05*	-1.68 (-2.52, -0.82)	0.58 ± 0.04*	1.76 (0.80, 2.69)
V1-RM (m/s)	0.54 ± 0.07	0.44 ± 0.07 *	-1.36 (-2.16, -0.54)	0.43 ± 0.05 *	1.69 (0.77, 2.59)

*p< 0.05 in comparison with the bilateral test.

SEE = standard error of the estimate; V40 = mean velocity at 40%1-RM; V70 = mean velocity at 70%1-RM; V1-RM = mean velocity at 100%1-RM; ES (95% CI) = effect size of the differences with respect to the bilateral test (with 95% confidence interval)

### Comparison of actual vs. estimated 1-RM

**Fig 3:**
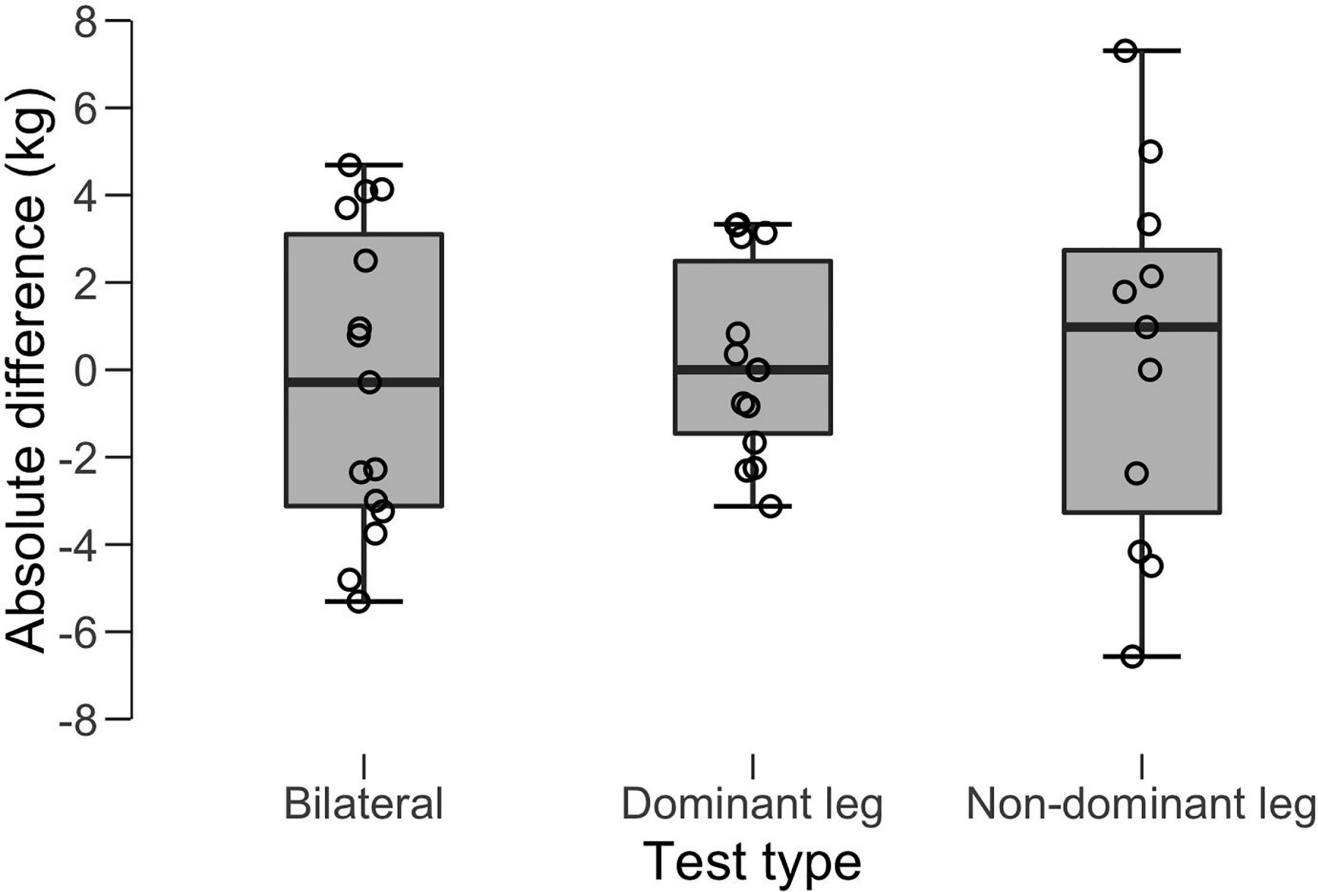
Boxplots with jitter points showing the absolute (kg) difference between actual and estimated 1-RM with bilateral (A), unilateral -dominant leg- (B) and unilateral -non-dominant leg- tests.

When comparing actual and estimated 1-RM, non-significant differences, with trivial to small ES were observed between the actual 1-RM and the 1-RM estimated using individual load-velocity relationships (Bilateral: ES = 0.02, 95% CI = -0.69, 0.73, p = 0.953; Dominant leg: ES = -0.25, 95% CI = -1.00, 0.48, p = 0.499; Non-dominant leg: ES = -0.33, 95% CI = -1.17, 0.51, p = 0.443). Actual and estimated 1-RM were very highly correlated as revealed by Pearson’s product-moment correlation coefficient (Bilateral: *r* = 0.96, SEE = 3.4kg, p <0.05; Dominant leg: *r* = 0.96, SEE = 2.2 kg, p <0.05; Non-dominant leg: *r* = 0.88, SEE = 3.6kg, p <0.05). See Fig 3 for more details.

## Discussion

This study aimed to analyze the goodness of fit of bilateral and unilateral load-velocity relationships in the leg extension exercise, and to analyze the differences between actual and estimated 1-RM. Specifically, results in our study showed that both bilateral and unilateral individual load-velocity relationships had similar levels of fit to what was previously investigated in different multi-joint exercises, with values of R^2^ higher than 0.93. Generalized load-velocity relationships showed weaker levels of agreement both in the bilateral and unilateral test (R^2^ = 0.52–0.58) and higher standard errors of the estimate (SEE = 10.3–10.7%1-RM) than those observed when computing individual equations. This is in line with previous research that showed that individual equations are more appropriate when analyzing the load-velocity relationships in different resistance exercises; thus, in order to calculate more accurate estimations of the 1-RM by measuring movement velocity, practitioners are encouraged to perform individual load-velocity relationships rather than using generalized equations [3,7,10,18].

Another relevant finding of the present study is that the goodness of fit was not significantly different between the bilateral and unilateral relationships (p> 0.05); however, significant, moderate to high differences were found in several parameters of the load-velocity relationship. Specifically, result in our study showed that unilateral relationships had lower intercepts in the Y-axis and, derived from that, lower mean velocities associated to low (40%1-RM), moderate (70%) and maximum (1-RM) loads. Previous research has observed that upper-body exercises produce lower mean velocities than lower-body exercises [5,6], and it was suggested that this might be due to the smaller muscle groups involved in the movement. Results in our study are in line with those investigations by showing that the unilateral leg extension exercise produces lower mean velocities at each %1-RM than the bilateral leg extension. However, other studies have observed that the levels of force, EMG activity and muscle coordination during a bilateral lower limb explosive contractions are significantly lower in comparison with unilateral efforts [19]. More research is needed to better understand the mechanisms of this bilateral deficit and its role in movement velocity at different loads during the leg extension exercise.

Finally, it was observed that the 1-RM estimated from individual load-velocity relationships was similar to the actual 1-RM, non-significant differences, with trivial to small ES between them (ES < 0.2, p> 0.05). However, given the width and location of the 95% confidence interval of the effect size, these results should be taken with precaution. For example, some studies have shown that V1-RM can remarkably vary between subjects, therefore producing high errors of the estimate in barbell exercises [6,20,21]. Thus, more studies are needed in order to confirm the accuracy of the load-velocity relationship in the leg extension exercise. Taken together, these results show that load-velocity relationships in a single-joint, machine-based exercise computed by measuring the mean vertical velocity of the weight-plates have similar levels of fit to traditional exercises and can provide accurate estimates of the 1-RM. This proof of concept could help researchers and strength and conditioning coaches who wish to study the load-velocity relationships in other popular machine-based exercises by using a simple smartphone application.

## Conclusions

There is a high association between load (in terms of %1-RM) and the mean vertical velocity of the weight plates both in the bilateral and unilateral leg extension exercise (R^2^ > 0.93). Also, it was shown that 1-RM can be accurately estimated by using the load-velocity relationships, as revealed by the small, non-significant differences observed in comparison with the actual 1-RM (ES < 0.2, p > 0.05). This proof of concept proved that load-velocity relationships can be measured in a machine-based, single-joint exercise by video-analyzing the vertical velocity of the weight plates using a smartphone application. To date, load-velocity relationships have been analyzed mostly in barbell exercises [1,5,6]. This study shows an alternative way to analyze the load-velocity relationships in machine-based exercises, like the biceps or hamstring curl, for example.
